# Seamless MEMS-INS/Geomagnetic Navigation System Based on Deep-Learning Strong Tracking Square-Root Cubature Kalman Filter

**DOI:** 10.3390/mi14101935

**Published:** 2023-10-15

**Authors:** Tianshang Zhao, Chenguang Wang, Chong Shen

**Affiliations:** The State Key Laboratory of Dynamic Measurement Technology, and The School of Instrument and Electronics, North University of China, Taiyuan 030051, China; sz202106044@st.nuc.edu.cn (T.Z.); wangchenguang@nuc.edu.cn (C.W.)

**Keywords:** deep self-learning, microelectromechanical system, cubature Kalman filtering, strong tracking filter, square-root filter, geomagnetic navigation, inertial navigation, integrated navigation

## Abstract

To suppress inertial navigation system drift and improve the seamless navigation capability of microelectromechanical system-inertial navigation systems/geomagnetic navigation systems (MEMS-INS/MNS) in geomagnetically unlocked environments, this paper proposes a hybrid seamless MEMS-INS/MNS strategy combining a strongly tracked square-root cubature Kalman filter with deep self-learning (DSL-STSRCKF). The proposed DSL-STSRCKF method consists of two innovative steps: (i) The relationship between the deep Kalman filter gain and the optimal estimation is established. In this paper, combining the two auxiliary methods of strong tracking filtering and square-root filtering based on singular value decomposition, the heading accuracy error of ST-SRCKF can reach 1.29°, which improves the heading accuracy by 90.10% and 9.20% compared to the traditional single INS and the traditional integrated navigation algorithm and greatly improves the robustness and computational efficiency. (ii) Providing deep self-learning capability for the ST-SRCKF by introducing a nonlinear autoregressive neural network (NARX) with exogenous inputs, which means that the heading accuracy can still reach 1.33° even during the MNS lockout period, and the heading accuracy can be improved by 89.80% compared with the single INS, realizing the continuous high-precision navigation estimation.

## 1. Introduction

As unmanned vehicles and other unmanned areas of research become more and more popular [1], a variety of high-precision navigation and positioning methods are also rapidly developing, such as global navigation satellite systems (GNSS), inertial navigation systems (INS), vision navigation systems, geomagnetic navigation systems (MNS), bionic navigation systems, and microelectromechanical navigation systems (MEMS), etc. These are more widely used navigation methods, and the characteristics of each navigation system and the respective strengths and weaknesses of the mutual coexistence of the systems are examined in [2,3,4,5,6].

This paper investigates an autonomous navigation system with unmanned capability applied in outdoor environments. Microelectromechanical systems-based inertial navigation (MEMS-INS) has the advantages of small size and strong anti-jamming, and it can maintain a short period of high-precision localization under autonomous use, but it has the fatal disadvantage of time-accumulated errors and cannot be used alone [7]. MNS is a passive system that utilizes the Earth’s magnetic field for positioning and navigation and has advantages such as no accumulation errors in time and distance. Therefore, the two navigation methods are complementary, and INS and MNS are combined together to suppress the drift of heading measurements. Florida Atlantic University [8] conducted research on INS/MNS information fusion to improve navigation accuracy. J.A. Barraza Madrigal implemented a 3D tracking method of the shoulder joint with respect to the thoracic cavity by using IMU/MARG integrated sensors and data fusion algorithms [9]. Due to the measurement errors of gyroscopes, accelerometers, and magnetometers inside the MIMU, many studies have proposed sensor fusion algorithms (SFAs) to accurately and robustly estimate the 3D orientation [10]. The above studies have provided some important insights into combined INS/MNS navigation. In this study, due to the complexity of the system as a nonlinear motion model, exploring how to incorporate auxiliary algorithms based on traditional filtering algorithms to optimize the fusion in order to reduce the error has been an important issue in combined navigation systems; therefore, we focused on the study of utilizing a variety of filtering schemes for more efficient fusion of INS/MNS information for more efficient fusion.

As a widely used optimization estimator, the Kalman filter (KF) is a customary filtering scheme to improve the accuracy of combinatorial navigation. However, when applied to motion nonlinear models, such as non-uniformly traveling carts or UAVs, it can cause the filter to be suboptimal or even divergent, and its performance is not satisfactory [11]. Therefore, a combined approach with the extended Kalman filter (EKF) and interactive multi-modeling is proposed to deal with the noise factor and to enable the use of highly dynamic models in non-motorized situations [12]. Since the EKF is sensitive to nonlinear errors and its linearization process introduces a significant truncation error, as well as a significant Jacobi matrix computation, three untraceable Kalman filters (UKFs) with different noise covariances are combined with interactive multi-models into an integrated framework to compensate for the filter divergence [13]. The cubature Kalman filter (CKF) utilizes the third-order spherical radial volume rule to make possible the numerical computation of multivariate moment integrals encountered in nonlinear Bayesian filters, and the CKF can be applied to solving an extensive range of nonlinear filtering problems, ranging from low to high dimensions [14]. Ref. [15] proposed an improved strong-tracking untraceable Kalman filter (ST-UKF) to resist the interference of motion modeling errors on combined INS/GNSS state estimation. The square root CKF (SRCKF) has shown excellent performance in non-Gaussian measurement noises and has received increasing attention [16]. However, in practical applications, due to the complexity of the system, there are more or less errors in the acquisition of the filter parameters, resulting in filter dispersion. In order to solve this problem, the concept of a strong tracking filter (STF) using the principle of vector orthogonality was proposed in [17], where an asymptotic cancellation factor is introduced to adjust the gain matrix over time for estimation. Based on the above research, this paper proposes to combine STF with SRCKF to develop a strong tracking SRCKF (ST-SRCKF) integrated filtering scheme for INS/MNS combined information fusion. The experimental results in this paper show that this filtering algorithm can improve the robustness of the combined navigation system, as well as reduce the number of unnecessary calculations and improve the computational efficiency.

Meanwhile, in practical applications, combined navigation is easily affected by external interference or inaccurate statistical information of sensor noise; for example, when a GPS-equipped ground vehicle or UAV passes through an environment with weak GPS signals, it will suffer from the problem of loss of lock, which leads to a drastic decrease in the navigation accuracy of the INS single-operating mode [18]. Similarly, geomagnetic navigation can be affected in strong magnetic environments; thus, solving the navigation disruption problem becomes more important. There is a growing interest in deep learning-related solutions, where an improved multilayer perceptron (MLP) artificial neural network directly relates INS velocity, angular rate, and acceleration to GPS position increments to predict velocity and position errors during GPS failures [19]. In recent years, deep learning has achieved satisfactory performance in time series prediction by integrating radial basis function neural networks (RBFNN) with time series to measure updates with prediction [20]. A recurrent neural network (RNN) was integrated with UKF to estimate and compensate for random drifts in gyroscopes [21]. By introducing a long- and short-term memory LSTM neural network to solve the problem of gradient vanishing and gradient explosion in RNNs during long-time sequences, the relationship between the internal gain and observations in combinatorial navigation is established to improve the error prediction accuracy [22]. In recent years, nonlinear autoregressive networks with exogenous inputs (NARX) have performed very well in data-driven metamodeling of nonlinear complex dynamic systems [23]. A nonlinear exogenous input autoregressive model for nonlinear systems based on neural network and time series analysis is proposed to predict the power generation of the photovoltaic module (PVM) for one month [24]. The above findings also provide an important guideline for the research ideas in this paper. The importance and innovation of this study lie in developing a seamless fusion strategy that combines the ST-SRCKF information fusion technique with the NARX neural network prediction tool applied to seamless combined INS/MNS navigation. The contributions of this paper are summarized as follows:

(1) In the IMU measurement unit, for the attitude angle information (pitch and roll angles) obtained from the gyroscope output solution, this paper fuses it with the additive counting data based on the quaternion solution method using the EKF to obtain more accurate and stable attitude information of pitch and roll angles. On the premise of the attitude information obtained by the above method, this paper proposes an ST-SRCKF algorithm to realize the fusion of INS information and MNS information and introduces an asymptotic factor to adjust the gain matrix over time for estimation so as to improve the robustness of the combined navigation system. The implementation utilizes singular value decomposition (SVD) to replace the original Cholesky decomposition process of SRCKF, which reduces the computational complexity and improves the numerical stability of the algorithm.

(2) A seamless fusion strategy is established to map the nonlinear relationship between INS inputs and heading observations during MNS outages. During the MNS availability time, the ST-SRCKF algorithm fuses the INS and MNS data to obtain accurate and reliable heading angle attitude information. Meanwhile, the NARX network is applied to train the INS data and MNS data to mine the relationship between INS parameters and heading observations. When the MNS is unavailable, the trained NARX predicts the lost observations during the MNS outage. Avoiding a single navigation situation ensures continuous navigation output with high accuracy.

The rest of the paper is organized as follows: Section 2 details the mathematical model of the combined INS/MNS navigation system and the proposed ST-SRCKF algorithm. Section 3 describes modeling the seamless fusion strategy over the NARX network in case of an MNS outage. Section 4 conducts experiments and simulations for validation. Section 5 concludes.

## 2. Inertial Navigation Systems/Geomagnetic Navigation Systems (INS/MNS) Navigation Model Based on Strongly Tracked Square-Root Cubature Kalman Filter (ST-SRCKF)

In combined navigation, INS is an essential part; in this paper, the EKF algorithm is utilized for the initial fusion of the INS inputs, i.e., gyroscope information and accelerometer information; the state quantity consists of the attitude quaternion and the gyroscope three-axis drift $\left[q_{0},q_{1},q_{2},q_{3},\varepsilon_{E},\varepsilon_{N},\varepsilon_{U}\right]\;$total of seven dimensions; and the accelerometer is used as a quantitative model to do the a posteriori estimation. This system resolves the carrier attitude information and has a better estimation for the traversing roll and pitching attitude information. The detailed settlement process can be found in [25]. However, due to the time drift of the INS, the obtained heading attitude information deviates greatly. As a complex nonlinear motion system, the deviation of the system model parameters leads to the filter performance degradation. In order to solve this problem, the concept of STF using the principle of vector orthogonality is introduced, and the asymptotic cancellation factor is introduced to adjust the gain matrix over time. In order to address the complexity of the computation of the mean-square error array $P_{k}$ of the CKF using the Cholesky decomposition, the singular value decomposition (SVD) is utilized instead of the original Cholesky decomposition process of the SRCKF, which effectively reduces the complexity of the computation. Based on the attitude angle information obtained from the INS solution, the proposed strongly tracked square-root cubature Kalman filter (ST-SRCKF) algorithm is used to incorporate the magnetometer data.

**Figure 1:**
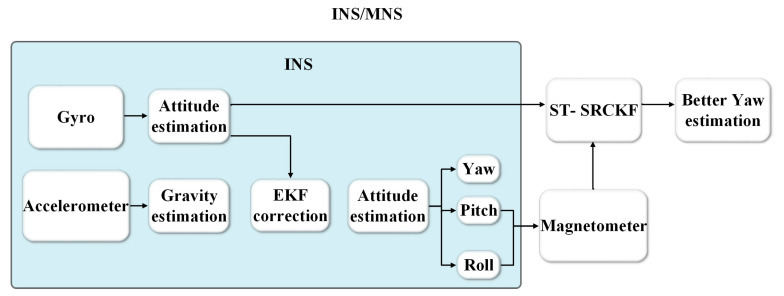
Block diagram of Inertial Navigation Systems/Geomagnetic Navigation Systems (INS/MNS) integrated navigation.

Taking the heading angle and gyro *Z*-axis bias of the gyro solution output as the state quantities and the heading angle compensated for tilt through the magnetometer data as the quantity measurement to do the a posteriori estimation, the optimal estimation of the heading attitude information is carried out by utilizing the proposed ST-SRCKF filtering algorithm, and the block diagram of the combined navigation is shown in Figure 1.

The equations of the state motion and measurement are expressed as

(1) $$\left[\begin{array}{c} \psi_{k}\\ b_{k} \end{array}\right]=\left[\begin{array}{cc} 1 & -T\\ 0 & 1 \end{array}\right]\left[\begin{array}{c} \psi_{k-1}\\ b_{k-1} \end{array}\right]+\left[\begin{array}{c} T\\ 0 \end{array}\right]\left[w_{k}\right]$$

(2) $$\left[\begin{array}{c} \psi_{m} \end{array}\right]=\left[\begin{array}{c} 1\;\;\;\;0 \end{array}\right]\left[\begin{array}{c} \psi_{k}\\ b_{k} \end{array}\right]$$

respectively, where $\psi_{k}$ represents the heading angle; $b_{k}$ is the gyroscope *Z*-axis offset; $w_{k}$ is the gyroscope-measured angular velocity; $\left[\begin{array}{cc} 1 & -T\\ 0 & 1 \end{array}\right]$ represents the state matrix $A_{k}$, and $\left[\begin{array}{c} 1\;\;\;\;0 \end{array}\right]$ is measurement matrix $H_{k}$; and *T* is the update period.

Inclination compensation of the INS output attitude by magnetometer data was used to obtain the actual observations

(3) $$\begin{array}{c} M_{y}=m_{x}*\sin\left(\theta \right)*\sin\left(\phi \right)+m_{y}*\cos\left(\theta \right)-m_{z}*\cos\left(\phi \right)*\sin\left(\theta \right)\\ M_{x}=m_{x}*\cos\left(\phi \right)+m_{z}*\sin\left(\phi \right)\\ Z_{k}=\psi =\mathrm{atan}2\left(M_{y},M_{x}\right) \end{array}$$

where ϕ, θ, and ψ represent the roll, pitch, and heading, respectively; and $m_{x}$, $m_{y}$, and $m_{z}$ are the magnetometer-measured three-axis data.

The entire process of the ST-SRCKF is divided into two parts: time update and observation update. The specific process is as follows:

(1) Time update:

➀ The square root of the covariance matrix is:

(4) $$\left[U,D,V\right]=\mathrm{svd}\left(P_{k-1}\right),\;\;P_{k-1}=U_{k-1}S_{k-1}V_{k-1}^{T}$$

➁ Calculate and propagate the volume points:

(5) $$X_{k/k-1}^{i}=A_{k}\left(U_{k-1}\sqrt{S_{k-1}}\xi_{i}+{\hat{x}}_{k-1}\right)$$

where $\xi_{i}=\sqrt{n}[1{]}_{i},\;i=1,2,\cdots \cdots ,n$, and n denote the state quantity dimension 2; [1] is the representative unit matrix; and ${\hat{x}}_{k-1}$ is the previous state estimate.

➂ Predict the state:

(6) $${\hat{x}}_{k/k-1}^{i}=\frac{1}{2n}\sum_{1}^{2n}\;X_{k/k-1}^{i}$$

➃ Calculate the a priori error covariance matrix:

(7) $$P_{k}^{-}=\frac{1}{2n}\sum_{i=1}^{2n}\;\left(X_{k/k-1}^{i}\right){\left(X_{k/k-1}^{i}\right)}^{T}-\left({\hat{x}}_{k/k-1}^{i}\right){\left({\hat{x}}_{k/k-1}^{i}\right)}^{T}+Q_{k}$$

➄ where $\lambda_{k}$ is the regulatory factor. The calculation formula is shown as follows:

(8) $$\lambda_{k}=max\left(\frac{\mathrm{tr}\left(W_{k}-H_{k}Q_{k}H_{k}^{T}-\beta R_{k}\right)}{\mathrm{tr}\left(H_{k}P_{k}^{-}H_{k}^{T}+Q_{k}\right)}\right)$$

(9) $$W_{k}=\left\{\begin{array}{c} \varepsilon_{k}\varepsilon_{k}^{T},k=0\\ \frac{\rho W_{k-1}+\varepsilon_{k}\varepsilon_{k}^{T}}{1+\rho },k\geq 1 \end{array}\right.,\varepsilon_{k}=Z_{k}-{\hat{y}}_{k}$$

where ρ is the forgetting factor and is generally chosen to be ρ=0.95; β is a weakening factor that takes the value β≥1; ${\;\varepsilon }_{k}$ is the innovation, the difference between the true measurement and the state estimate.

➅ The modified error covariance matrix:

(10) $$P_{k|\lambda }^{-}=\lambda_{k}A_{k}P_{k}^{-}A_{k}^{T}+Q_{k}$$

(2) Update the observation:

➀ The square root of the covariance matrix $P_{k|\lambda }^{-}$:

(11) $$\left[U,D,V\right]=\mathrm{svd}\left(P_{k|\lambda }^{-}\right),\;\;P_{k|\lambda }^{-}=U_{k|\lambda }S_{k|\lambda }V_{k|\lambda }^{T}$$

➁ Calculate and propagate the volume points:

(12) $$y_{k}^{i}=H_{k}\left(U_{k|\lambda }\sqrt{S_{k|\lambda }}\xi_{i}+{\hat{x}}_{k/k-1}^{i}\right)$$

➂ Predict the measurement:

(13) $${\hat{y}}_{k}=\frac{1}{2n}\sum_{i=1}^{2n}\;y_{k}^{i}$$

➃ Calculate the observed predicted covariance and cross-covariance:

(14) $$\begin{array}{c} P_{k}^{y}=\frac{1}{2n}\sum_{i=1}^{2n}\;\left(y_{k}^{i}\right){\left(y_{k}^{i}\right)}^{T}-\left({\hat{y}}_{k}\right){\left({\hat{y}}_{k}\right)}^{T}+R_{k}\\ P_{k}^{xy}=\frac{1}{2n}\sum_{i=1}^{2n}\;X_{k}^{i}{\left(y_{k}^{i}\right)}^{T}-\left({\hat{x}}_{k/k-1}^{i}\right){\left({\hat{y}}_{k}\right)}^{T} \end{array}$$

➄ Calculate the Kalman gain, state update, and corresponding error covariance matrix:

(15) $$K_{k}=P_{k}^{xy}{\left(P_{k}^{y}\right)}^{-1}$$

(16) $${\hat{x}}_{k}={\hat{x}}_{k/k-1}^{i}+K_{k}\varepsilon_{k}$$

(17) $$P_{k}=P_{k}^{-}-K_{k}P_{k}^{y}K_{k}^{T}$$

**Figure 2:**
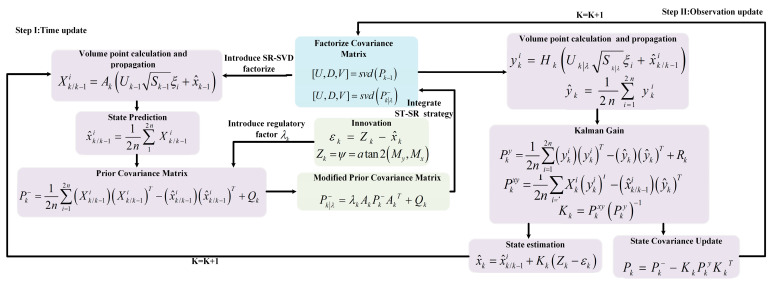
Block diagram of the Strongly Tracked Square-Root Cubature Kalman Filter (ST-SRCKF) algorithm.

The proposed combined navigation algorithm ST-SRCKF is essentially an iteration of the two processes of temporal update and measurement update, which is illustrated in Figure 2. In this paper, the initial estimates for $x_{0}$ and $P_{0}$ are provided so that the state estimates ${\hat{x}}_{k}$ can be computed recursively.

## 3. Seamless Fusion Strategy Based on Nonlinear Autoregressive with Exogenous Input (NARX)

### 3.1. Nonlinear Autoregressive Networks with Exogenous Input

Using a large amount of historical data to predict the future structural dynamic response effectively solves the problem of navigational disruption. The NARX neural network is based on a nonlinear autoregressive neural network with exogenous inputs. Compared with autoregressive and moving average models, NARX can capture the nonlinear relationship of the time series, which makes the model better adapted to the actual problem in complex environments, and its effectiveness in structural dynamic modeling has been proved in research [26]. The NARX network can discover long-term dependencies in the data through embedded input memory and is more compatible with gradient descent learning algorithms with faster convergence and better generalization than, e.g., LSTM recurrent neural networks with state variables [27]. 

For discrete time series, NARX can build the time series:

(18) $$y_{t}=f\left(u_{t},y_{t}\right),$$

(19) $$\left\{\begin{array}{c} u(t)={\left[\left(t-n_{ex}\Delta t\right),\cdots u\left(t-2\Delta t\right),u\left(t-\Delta t\right),u\left(t\right)\right]}^{T}\\ y(t)={\left[\left(t-n_{in}\Delta t\right),\cdots y\left(t-2\Delta t\right),y\left(t-\Delta t\right)\right]}^{T} \end{array}\right..$$

where Δt is the time step, $u\left(t\right)$ and $y\left(t\right)$ are referred to as internal and external inputs, $n_{ex}$ and $n_{in}$ are the number of lags in $u\left(t\right)$ and $y\left(t\right)$, respectively, and f is the right-to-left nonlinear basis function mapping data.

Replacement of the Equation (18) f function with the NARX network model

(20) $$\begin{array}{c} \hat{y}\left(t\right)=f\left(u\left(t\right),y\left(t\right)\right)\\ =h_{n+1}\left(W_{Ln+1,n}h_{n}\left(\cdots h_{2}\left(W_{L2,1}h_{1}\left(W_{I1,1}u\left(t\right)+b_{1}+W_{L1,n+1}y\left(t\right)\right)+b_{2}\right)\cdots \right)+b_{n+1}\right). \end{array}$$

$\hat{y}\left(t\right)$ is the model output; h,W and b are the activation function, the weight matrix (I denotes the input, and L denotes the number of network layers), and the deviation matrix constituting hypothesis f, respectively. After the number of hidden layers and neurons per layer are determined for the network structure, the performance function can be minimized by backpropagation with a gradient descent algorithm to iterate the network structure’s weight matrix and deviation matrix parameters.

### 3.2. Seamless Fusion Structure Based on NARX-Integrated ST-SRCKF

Since the combined INS/MNS navigation system is a nonlinear complex system, its combined mathematical model loses its accuracy when encountering an MNS outage situation. The proposed seamless fusion structure is precisely based on NARX to build the $O_{INS}-Yaw_{z_{k}}$ relational model, i.e., the relational model of specific force, angular velocity, and heading attitude information. It replaces the out-of-alignment mathematical model with the constructed relational model between the INS output information and the real measure $z_{k}$. This provides important insights for better handling of complex nonlinear systems. 

The input and output can be expressed as

(21) $$\mathrm{Input}:[w_{x}^{b},w_{y}^{b},w_{z}^{b},f_{x}^{b},f_{y}^{b},f_{z}^{b}]\;\mathrm{Ouput}:[{\mathrm{Yaw}}_{z_{k}}]$$

where $w_{x}^{b},w_{y}^{b},w_{z}^{b}$ represent the angular velocity of the tri-axial measured by the gyroscope; $f_{x}^{b},f_{y}^{b},f_{z}^{b}$ represent the tri-axial specific force measured by the accelerometer; and ${\;\mathrm{Yaw}}_{z_{k}}$ represents the measured heading angle.

The proposed NARX combined with the ST-SRCKF seamless fusion navigation structure is a two-mode structure. When MNS is available, the seamless fusion navigation strategy runs in training mode. The attitude information output from the INS solution is fused with the heading attitude information provided by the MNS in the ST-SRCKF to output the optimal estimate of the heading angle. Meanwhile, IMU raw and INS/MNS data are continuously fed into the NARX-based network model for training over a long period, iterating the network structure’s weight matrix and deviation matrix parameters to form a reliable $O_{INS}-{\;\mathrm{Yaw}}_{z_{k}}$ input-output relationship model.

**Figure 3:**
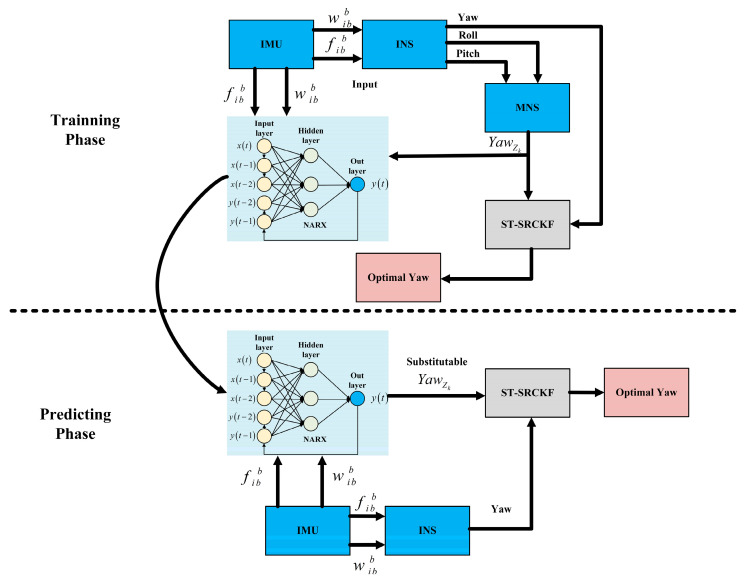
Seamless fusion structure based on NARX- integrated ST-SRCKF.

When the MNS outage is unavailable, the seamless fusion structure goes into prediction mode. The current IMU data are used as input, and a trained NARX network model is utilized to predict the pseudo-heading attitude quantity, which continues to be incorporated into the ST-SRCKF as the measurement information along with the attitude information provided by the IMU, thus obtaining the optimal estimation of the heading angle. The NARX-ST-SRCKF seamless fusion navigation structure is designed to ensure that the navigation accuracy does not deteriorate drastically in the event of a loss of the MNS signal. The NARX-ST-SRCKF seamless INS/MNS fusion navigation structure is designed to ensure that the navigation accuracy does not degrade dramatically when the MNS signal is lost, as shown in Figure 3. 

Combining neural networks with superior predictive capabilities with improved Kalman filtering algorithms can lead to a generalized and seamless fusion strategy that can be extended to other potential future applications with multiple sensor combinations. Examples include: monocular visual-inertial-liDAR simultaneous localization-fusion, a three-level multisensor fusion system that can achieve robust state estimation and globally consistent mapping in perceptually degraded environments [28]; convolutional neural network (CNN)-based faster regions with CNN (faster R-CNN) and you only look once (YOLO) V2 are investigated for improving the recognition technique of in-vehicle monocular cameras for designing preventive autonomous driving systems [29]; the uncertainty function is approximated using a radial basis function neural network (RBFNN). This means that vehicle platoon practical consensus can be achieved under the Zeno-free adaptive event-triggered control scheme, making connected automated vehicles’ platoon reach the string stability [30].

## 4. Experimental Results and Analysis

**Figure 4:**
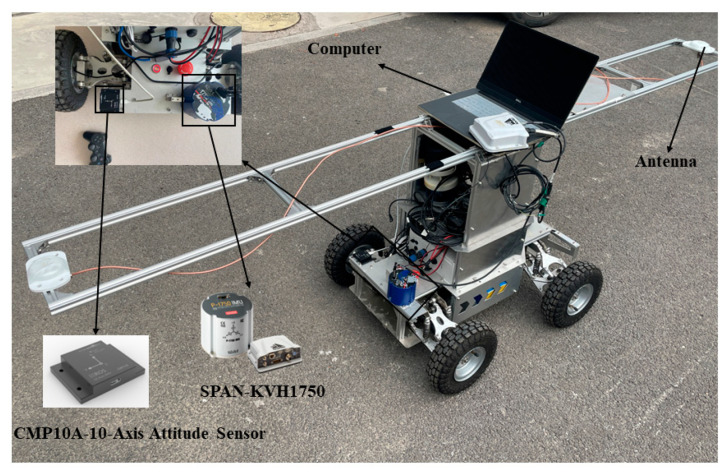
Experimental equipment.

To verify the effectiveness of the proposed seamless fusion structure based on NARX- integrated ST-SRCKF, we self-designed and built an unmanned vehicle platform, and the platform is equipped with a CMP10A-10-axis attitude sensor. Figure 4 shows the experimental setup and hardware structure. A three-axis geomagnetic sensor and IMU were integrated into the 10-axis attitude measurement device. The sensor details are shown in Table 1. A computer was responsible for receiving and displaying the experimental data and running the program to achieve the fusion process of the heading angle. The reference heading angle of the test system comes from a high-precision compact optical fiber integrated navigation system (Model: SPAN-KVH1750) and its heading accuracy and sampling frequency could reach 0.035° (root mean square, RMS). To verify the superiority of the algorithm, experiments were conducted, and eight representative methods were proposed for comparison.

(1) Method 1: Reference system.
(2) Method 2: Pure INS algorithm.
(3) Method 3: Pure Mag algorithm.
(4) Method 4: EKF INS/MNS algorithm.
(5) Method 5: CKF INS/MNS algorithm.
(6) Method 6: ST-EKF INS/MNS algorithm.
(7) Method 7: ST-SRCKF INS/MNS algorithm.
(8) Method 8: NARX-ST-SRCKF INS/MNS algorithm.

The test results are analyzed later in this section.

**Table 1:** Sensor details.

Sensors	Parameter	Value
SPAN-KVH1750	Heading angle accuracy Frequency	0.035° 100 HZ
CMP10A-10Axis Attitude Sensor	Accelerometer resolution Gyroscope resolution Magnetometer resolution	0.0005 (g/LSB) 0.061 (°/s) 0.0667 mGauss/LSB

### 4.1. Verification of the ST-SRCKF Algorithm

**Figure 5:**
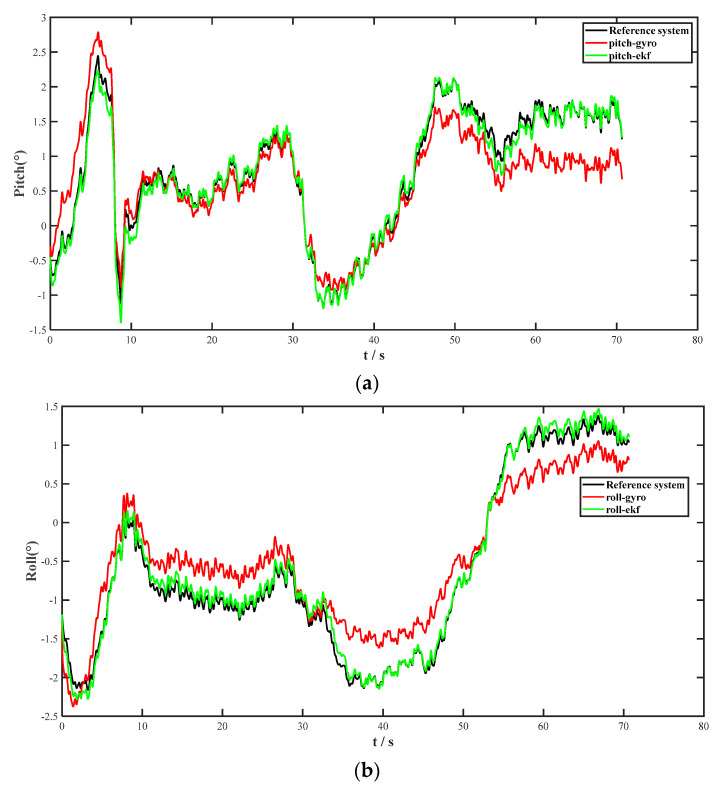
(**a**) Pitch angle in INS. (**b**) Roll angle in INS.

The test was conducted on 20 May 2023, at 12.40 p.m. at 3 College Road, Taiyuan, China (112.45° E, 38.02° N). The CMP10A-10-axis attitude sensor was mounted on the UV along with the reference system. In the initial stage of the experiment, the EKF fusion performance of the pitch and roll information of the IMU (three-axis gyroscope and three-axis accelerometer) was verified first, and then the EKF algorithm fusion process was performed for the collected three-axis gyroscope and three-axis accelerometer data. The comparison effect is shown in Figure 5a,b. The pitch and roll output accuracy and stability had a significant corrective effect.

**Figure 6:**
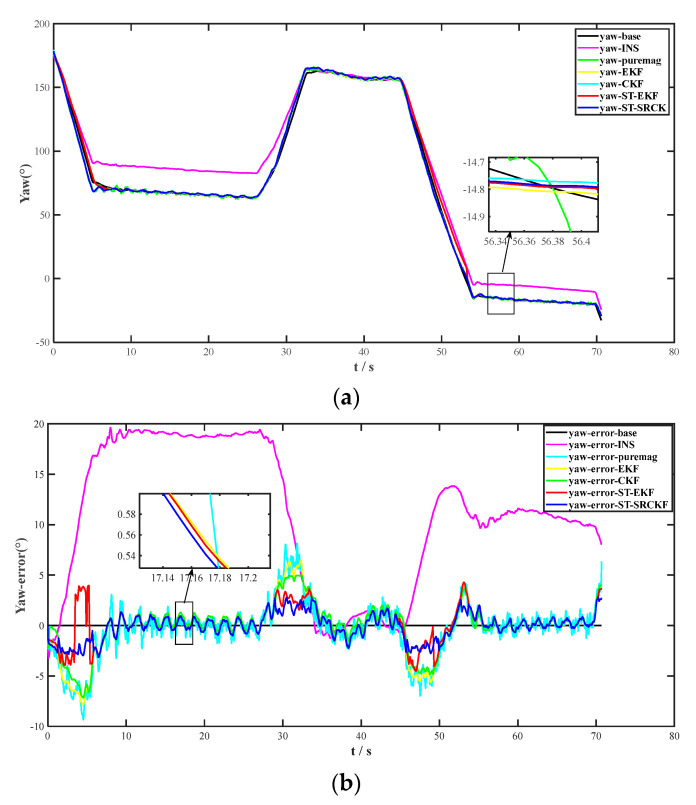
(**a**) Comparative analysis of the heading of the seven methods. (**b**) Comparative analysis of the heading error of the seven methods.

Under the normal geomagnetic condition, the tri-axial magnetometer data collected by the device were fused with the experimental attitude output results in the initial stage using four comparative fusion algorithms. To evaluate the performance of the heading fusion strategy, the heading and heading error curves of each of the seven methods were plotted, as shown in Figure 6a,b, which were used for comparison.

Method 2 is accompanied by the gradual accumulation of time errors, decreasing measurement accuracy. As shown in Figure 6b, the magnetometer is susceptible to the influence of other electronic devices, and in Method 3, the heading angle error is highly volatile. Method 4 makes an obvious improvement in heading accuracy due to the fusion of geomagnetic data. However, as a complex nonlinear motion system, the heading accuracy of the method is lower and easy to diverge compared with the nonlinear fusion algorithm of Method 5; at the same time, the deviation of the system model parameters will result in the performance of the filter being degraded. Therefore, Method 6 introduces the concept of STF, which utilizes the fading factor to adjust the gain matrix over time. Method 7 introduces square root filtering based on Method 6 and replaces the Cholesky decomposition with SVD decomposition, greatly reducing computational complexity.

The results show that the method enhances the robustness of the combined navigation system, significantly improves the performance of heading angle measurement, reduces the computational complexity, and improves the numerical stability of the algorithm. To further illustrate the effectiveness of the algorithms in this paper, the statistical characteristic values of the six algorithms are summarized in Table 2. Compared with Method 2, Method 4 improves heading accuracy by 80.90%, Method 5 improves heading accuracy by 82.98%, Method 6 improves heading accuracy by 87.35%, and Method 7 improves heading accuracy by 90.10%. Among them, Method 7 proposed in this paper has a higher performance in all numerical characteristics, and its adaptability and robustness of fused heading angles are better than other fusion algorithms.

### 4.2. Verification of the Seamless Fusion Structure

**Figure 7:**
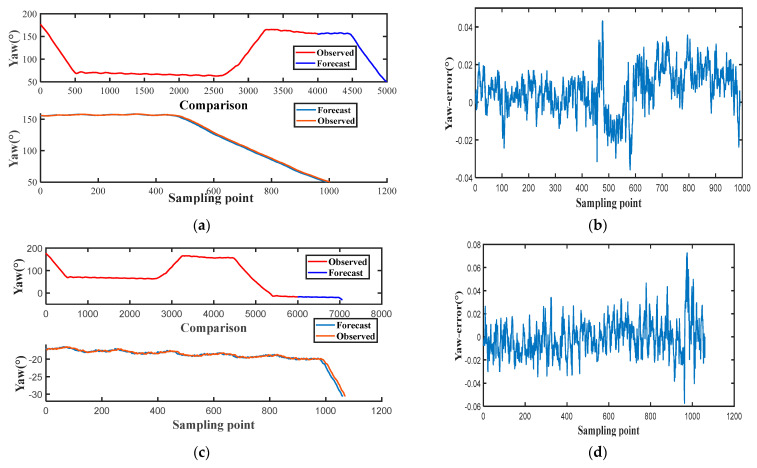
(**a**) Training and validation analysis based on the first phase of NARX. (**b**) Phase I prediction and observation error analysis. (**c**) Training and validation analysis based on the second phase of NARX. (**d**) Second-stage prediction and observation error analysis.

During geomagnetic disturbances, to validate the effectiveness of the proposed NARX integrated ST-SRCKF seamless fusion structure, data collected by the device are divided into two phases, and two sets of training predictions are performed using the NARX network. The hardware device used for its training network is a NVIDIA GeForce 940MX. In the first stage, the first 4000 sets of sampled data points were used to train the model, and the last 1000 sets of sampled data points were used to predict and test the model. In the second stage, the first 6000 sets of sampled data points were used to train the model, and the remaining data points were used to predict and test the model. This grouping is to cover the basic motion states of the cart and, at the same time, to train the network model more efficiently, and the results of the training and validation analysis are shown in Figure 7a–d.

**Figure 8:**
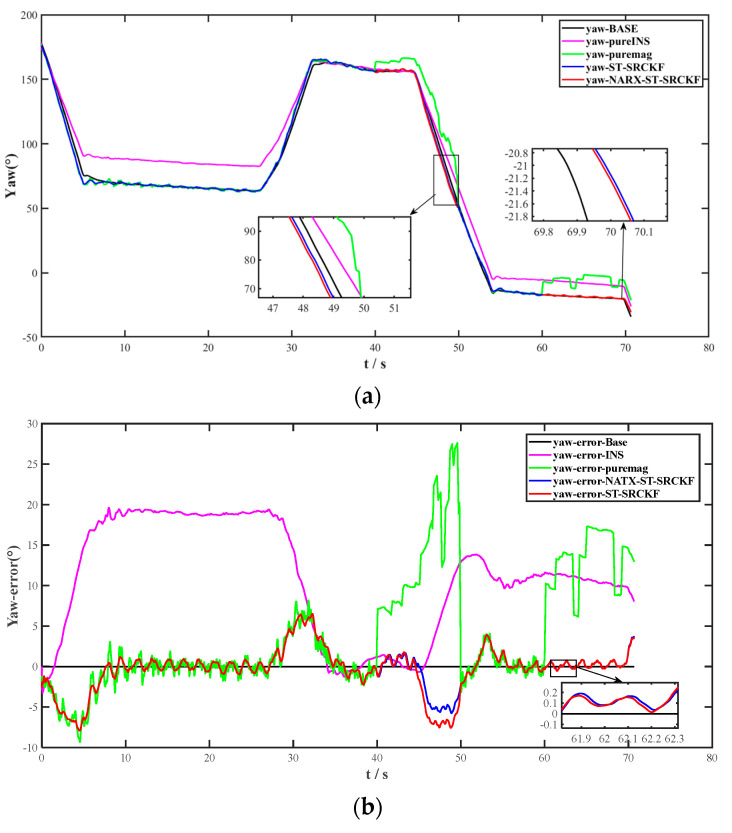
(**a**) Analysis of the heading angles of the five methods during periods containing geomagnetic interruptions. (**b**) Analysis of the heading angle errors of the five methods during the period containing the geomagnetic interruption.

The data predicted in these two segments will replace the missing data during the MNS outage. During the MNS normal period, Method 7 was used to process the data; during the MNS outage, Method 8 was used to process the data predicted by the model. In order to evaluate the performance of the seamless fusion strategy in the case of geomagnetic loss of lock, the heading and heading error curves of the five methods were plotted, as shown in Figure 8a, and the heading angle error analysis curves were plotted for comparison, as shown in Figure 8b.

During interruptions, the geomagnetic sensors are subjected to strong magnetic interference, increasing the magnetometer output error. In contrast, the INS error gradually increases over time, leaving only the INS available for heading analysis, and the seamlessly fused structural prediction model of the NARX-integrated ST-SRCKF seamlessly bridges the interruption gap. Table 3 shows the comparative analysis of the statistical characteristic values of Method 8 with the pure INS and pure mag. The results show that in the case of MNS interruption, the seamless fusion structure of NARX-integrated ST-SRCKF can compensate for the resulting loss of heading accuracy in the case of geomagnetic interruptions and ensures the high-precision output of navigation.

## 5. Conclusions

This study aims to address the problem of error accumulation in single navigation and the problem of fusion optimization and disruption in combined navigation. Therefore, this paper proposes a new method for a seamless fusion of INS/MNS navigation systems based on the NARX integrated ST-SRCKF algorithm, with some effects of suppressing the INS error and achieving high robustness of the navigation system and low computational complexity. During the MNS data-loss process, the proposed seamless fusion structure can automatically correlate the current input information with the historical model information to compensate for the resulting loss of heading accuracy. Field experiments and simulations are conducted to verify the proposed method’s performance.

The experimental results show that the effectiveness of the fusion algorithm of Method 7 is verified in a magnetically normal environment. The heading accuracy of Method 4 is improved by 80.90%, Method 5 is improved by 82.98%, Method 6 is improved by 87.35%, and Method 7 is improved by 90.10% compared with that of Method 2. In the magnetic anomaly environment experiment, Method 8 improved the heading accuracy by 89.80% compared with the pure INS Method 2. 

The results show that the designed seamless fusion strategy based on NARX network-assisted ST-SRCKF effectively estimates the inherent nonlinear relationship between the output of the inertial navigation system and the heading information and provides accurate navigation information when the MNS is interrupted. However, novel fusion filtering algorithms need to be explored in the future to minimize navigation errors further, designing fault detection schemes for autonomous detection in terms of active sensing of navigation system faults, and exploring deep learning networks with higher prediction accuracy in terms of interruption prediction compensation.

## Figures and Tables

**Table 2:** Median, max, min, and RMS of the yaw obtained by the six methods in magnetic normality experiment (units: °).

Method	Median	Max	Min	RMS	Improvement (RMS)
Method 2 Method 3	11.16 0.02	19.61 8.16	0.0021 8 × 10^−5^	13.04 2.78	\ \
Method 4	0.04	6.41	5 × 10^−5^	2.49	80.90%
Method 5	0.16	4.97	10^−4^	2.22	82.98%
Method 6 Method 7	0.11 0.03	4.29 2.80	7 × 10^−5^ 7 × 10^−5^	1.65 1.29	87.35% 90.10%

**Table 3:** Median, max, min, and RMS of the yaw obtained by the three methods in magnetic anomaly experiment (units: °).

Method	Median	Max	Min	RMS	Improvement (RMS)
Method2 Method3	11.16 0.55	19.61 17.7	0.0021 8 × 10^−5^	13.04 6.50	\ \
Method8	0.01	3.31	2 × 10^−5^	1.33	89.80%

## Data Availability

The raw data required to reproduce these findings cannot be shared at this time as the data also form part of an ongoing study.
